# Efficacy and Safety of Perioperative Immunotherapy for Patients with Non-Small Cell Lung Cancer: A Systematic Review and Network Meta-Analysis

**DOI:** 10.3390/curroncol32030184

**Published:** 2025-03-20

**Authors:** Zhijuan Du, Siyuan Chen, Yuhui Qin, Yahui Lv, Xiangyu Du, Heying Yu, Zhefeng Liu

**Affiliations:** 1 Medical School of Chinese PLA, Beijing 100853, China; 2 Department of Medical Oncology, The First Medical Center of Chinese PLA General Hospital, Beijing 100853, China; 3 Department of Medical Oncology, The Third Medical Center of Chinese PLA General Hospital, Beijing 100089, China; 4 Department of Medical Oncology, Senior Department of Oncology, The Fifth Medical Center of Chinese PLA General Hospital, Beijing 100039, China

**Keywords:** non-small cell lung cancer, immune checkpoint inhibitor, perioperative immunotherapy, Bayesian network meta-analysis

## Abstract

**Background:** The objective of this study is to indirectly compare the efficacy and safety of all currently available neoadjuvant chemoimmunotherapy and perioperative chemoimmunotherapy in randomized controlled trials (RCTs) involving patients with resectable non-small cell lung cancer (NSCLC) to identify optimal treatment regimens. **Methods:** Eligible studies evaluating neoadjuvant chemoimmunotherapy and perioperative chemoimmunotherapy-based regimens in resectable NSCLC patients were included. Clinical outcomes were extracted for event-free survival (EFS) and overall survival (OS), as well as the incidence of pathological complete response (pCR), major pathological response (MPR), any-grade adverse events (AEs), and treatment-related adverse events (TRAEs) in the Bayesian framework. A subgroup analysis of EFS was conducted according to PD-L1 expression, histological type and reaching pCR or not. **Results:** We selected eight RCTs involving 3113 patients. Our analysis found no significant differences between perioperative immunotherapy and neoadjuvant immunotherapy in terms of MPR (RR 0.72, 95% CI 0.39 –1.3), pCR (RR 0.73, 95% CI 0.24–2.3), EFS (HR 0.95, 95% CI 0.56–1.7), and OS (HR 95% CI 3.9–4.2). Subgroup analyses revealed that neoadjuvant immunotherapy demonstrated superiority in the programmed death-ligand 1 (PD-L1) high-expression cohort, the non-squamous cell carcinoma cohort, and the non-smoking cohort. Conversely, perioperative immunotherapy ranked first in the PD-L1 low-expression cohort, squamous cell carcinoma cohort, and non-pCR cohort. **Conclusions:** Our findings indicate that neoadjuvant immunotherapy and perioperative immunotherapy exhibit comparable efficacy in patients with NSCLC. These results provide valuable evidence for guiding the treatment of patients with resectable NSCLC.

## 1. Introduction

Lung cancer is the primary factor leading to cancer-related deaths in both China and globally, accounting for around 20% of all cancer-related fatalities [1,2]. Non-small cell lung cancer (NSCLC) is the most common kind of lung cancer, making up over 85% of all cases [3]. Immune checkpoint inhibitors (ICIs) have demonstrated promising efficacy and safety profiles in the treatment of lung cancer, particularly in the management of NSCLC [4,5,6,7,8].

The NADIM trial (NCT03081689) represented a notable advancement in evaluating the viability of neoadjuvant chemoimmunotherapy. Despite its single-arm design, the study reported impressive outcomes, including a major pathological response (MPR) rate of 83%, and a pathological complete response (pCR) rate of 63% [9,10]. Following the NADIM II trial (NCT03838159), it provided further evidence by demonstrating that neoadjuvant chemoimmunotherapy, compared with neoadjuvant chemotherapy, notably enhanced the pCR rate and MPR rate (7% vs. 37%; 14% vs. 52%) among patients diagnosed with resectable NSCLC [11,12]. The CheckMate 816 trial (NC02998528), the inaugural phase III clinical trial of neoadjuvant chemoimmunotherapy, also affirmed its efficacy by showing significantly increases in the MPR rate (36.9% vs. 8.9%) [13]. Furthermore, several recent randomized clinical trials, including RATIONALE-315 (NCT04379635) [14], NEOTORCH (NCT04158440) [15], CheckMate77T (NCT04025879) [16], AEGEAN(NCT03800134) [17], KEYNOTE-671 (NCT03425643) [18], and TD-FOREKNOW (NCT04338620) [19], have reinforced the potential of neoadjuvant chemoimmunotherapy. The MPR rates documented in these studies varied, ranging from 30.2% to 65.1%, with pCR rates ranging from 17.2% to 40.7%. In addition to the clinical trial outcomes associated with neoadjuvant chemoimmunotherapy, perioperative immunotherapy for NSCLC has also shown promising efficacy and safety profiles in clinical trials. One study found that a pCR was observed in 37% of patients in the perioperative immunotherapy group, compared to 7% in the chemotherapy group. Kaplan–Meier estimates of 24-month PFS were 67.2% vs. 40.9%, and 24-month OS was 85.0% vs. 63.6% [11]. Other studies have also observed significant benefits of perioperative immunotherapy compared to chemotherapy [17,18]. While perioperative immunotherapy has shown promising results in NSCLC treatment, there remains contentious debate regarding the effects and safety of neoadjuvant immunotherapy-based therapy compared to the combination of neoadjuvant and adjuvant immunotherapy-based therapy in NSCLC patients. Therefore, this network meta-analysis aims to indirectly compare neoadjuvant immunotherapy-based therapy with the combination of neoadjuvant and adjuvant immunotherapy-based therapy, both of which share the same control group intervention, to provide clarity on this matter. This review was performed following the PRISMA checklist.

## 2. Methods

We adhered to the Preferred Reporting Items for Systematic Reviews and Meta-Analyses criteria, incorporating an extension for Network Meta-Analysis (NMA) [20]. The study protocol was officially registered with PROSPERO (CRD42024504522).

### 2.1. Search Strategies

We included all relevant studies from databases including PubMed, Embase, MEDLINE, Cochrane Central Register of Controlled Trials databases, and ClinicalTrials.gov up to 27 November 2023. Additionally, we screened abstracts from prominent conferences such as the American Society of Clinical Oncology (ASCO), European Society of Medical Oncology (ESCO), World Conference on Lung Cancer (WCLC), American Association for Cancer Research (AACR), and European Lung Cancer Congress (ELCC). The detailed strategies are provided in Supplementary Tables S1–S3.

### 2.2. Inclusion and Exclusion Criteria

Based on the PICOS Framework, the inclusion and exclusion criteria for selecting papers are outlined as follows:

Inclusion Criteria: (I) Studies enrolling patients diagnosed with NSCLC, confirmed through histological or cytological examination; (II) Studies directly comparing neoadjuvant immunotherapy plus chemotherapy with neoadjuvant chemotherapy; (III) Studies directly comparing perioperative immunotherapy plus chemotherapy with perioperative chemotherapy; (IV) Published randomized controlled trials (RCTs), including open-label, double-blind, and triple-blind trials; (V) Studies reported one or more of the detailed outcomes, including MPR, pCR, event-free survival (EFS), overall survival (OS), any-grade adverse events (AEs) and treatment related adverse events (TRAEs).

Exclusion Criteria: (I) Single-arm experiments; (II) Studies reporting endpoints of patients with NSCLC based solely on subgroup findings; (III) Meta-analyses, reviews, letters, surveys, case reports, and book chapters; (IV) Studies involving nonhuman subjects; (V) Incomplete studies.

### 2.3. Data Extraction

Two authors autonomously gathered the data, and any inconsistencies were rectified through deliberations with the remaining authors. The data extracted from each article include the trial name, first author, publication sources, year of publication, trial phase, National Clinical Trials identification number, sample size, patients’ age and gender, performance status, pathological stage, smoking status, histological features, PD-L1 expression, and geographic region. Furthermore, we extracted clinical outcomes such as hazard ratios (HRs) along with their corresponding 95% confidence intervals (95% CIs) for EFS and OS. We also collected data on the incidence of PCR, MPR, any-grade adverse events (AEs), treatment-related adverse events (TRAEs), and AEs and TRAEs of grade 3 or higher.

### 2.4. Quality Assessment

The quality of the included studies was assessed using the Cochrane Risk of Bias Tool (version 2.0) for RCTs [21]. Potential biases were classified as “high risk”, “low risk” or “unclear” across the following domains: (I) random sequence generation; (II) allocation concealment; (III) blinding of participants and personnel; (IV) blinding of outcome assessment; (V) incomplete outcome data; (VI) selective outcome reporting; and (VII) other biases. Two authors independently assessed the potential for bias in each study, with any discrepancies resolved through consensus and arbitration by a panel of adjudicators.

### 2.5. Statistical Analysis

The study retrieved hazard ratios (HRs) and their accompanying 95% confidence intervals (95% CIs) for each time-to-event endpoint. The HR compares the rate of an event occurring over time between two groups. The risk ratio (RR) compares the probability of an event occurring between two groups over a fixed period. HR is used for time-to-event outcomes like EFS and OS because it accounts for the rate of events over time, while RR is used for categorical outcomes like MPR and pCR because it compares the probability of an event occurring at a specific time point. The overall effect was evaluated using a random-effects model based on inverse variance. The data collection process for categorical endpoints involved recording the number of events for each group. In situations where the exact number of events was not provided, it was calculated based on the percentage of cases with an event. To minimize bias from varying follow-up durations, we included the most recent and complete datasets from each study, prioritizing those with the longest follow-up. A sensitivity analysis was also conducted to assess the robustness of the results, accounting for differences in follow-up durations. The study employed a Mantel–Haenszel random-effects model to determine the odds ratios (ORs) along with their corresponding 95% CIs. The presence of statistical heterogeneity among the research included in the analysis was evaluated using the Chi-squared test (X^2^) and I^2^ statistics. A fixed-effects model was selected for pooling estimates if the *p*-value for X^2^ was greater than 0.1 and I^2^ was less than 50%. Conversely, a random-effects model was used if either the *p*-value for X^2^ was less than 0.1 or I^2^ was greater than or equal to 50% [22]. A statistically significant level of heterogeneity was considered if I^2^ was greater than 50% or the *p*-value for X^2^ was less than 0.1. No statistically significant difference was deemed present when the 95% CI for indirect comparison encompassed 1. The ranking of treatment effects for all comparison variables (MPR, pCR, EFS, OS, AEs, and TRAEs) was established based on potential outcomes. Three Markov chains, each with different initial values, were operated simultaneously for 100,000 iterations for each outcome. A thinning interval of 10 was used, and the early 10,000 iterations were discarded as burn-ins. The NMA evaluated the probability of each therapy being the most effective among all therapies by comparing the effects of all therapies in each iteration and calculating the proportion of times each therapy was ranked first across all iterations. Publication bias was evaluated using funnel plots and also Egger and Begg tests. The statistical analyses were conducted using R (version 4.3.1) and R Studio software (4.3.1). The Gemtc package (1.0-2) was employed for the statistical analysis.

## 3. Results

### 3.1. Included Studies

**Figure 1:**
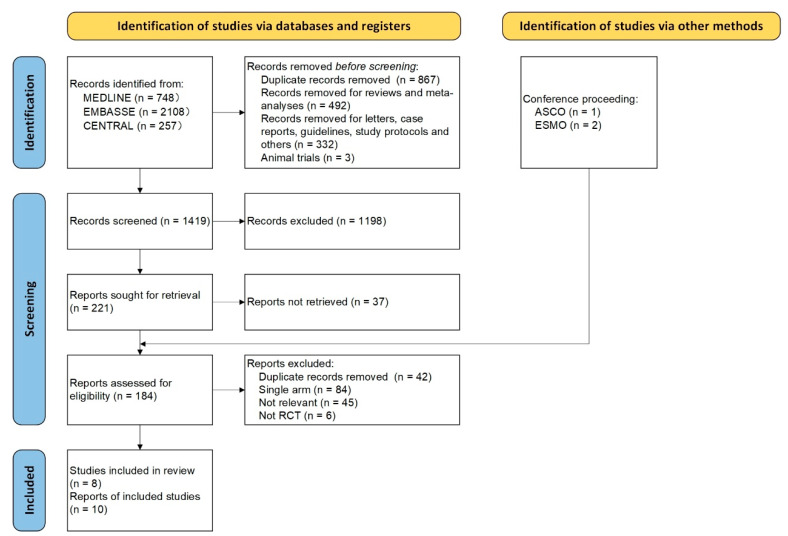
PRISMA flow diagram of the study.

We initially identified 3113 associated publications from databases including PubMed, Embase, MEDLINE, Cochrane Central Register of Controlled Trials databases, and ClinicalTrials.gov, which also included conference abstracts. After removing duplicates (876 records) and articles such as reviews, meta-analyses, letters, case reports, guidelines, study protocols, and animal trials (827 records), 1419 articles remained for screening. Following the evaluation of titles, 1198 articles were disqualified for having unrelated subjects. Then, after carefully reading the abstracts of the 221 articles, 37 records were excluded. After a thorough examination of the conference abstracts and full-text articles, ten articles were ultimately deemed eligible. In the end, this NMA contained eight RCTs. Figure 1 displays the selection process for the items that were searched. Of the eight RCTs, six focused on perioperative immunotherapy versus chemotherapy (involving 3003 patients), while two examined neoadjuvant immunotherapy (involving 446 patients). Of the six RCTs involving perioperative intervention, two investigated chemo-nivolumab followed by nivolumab, one examined chemo-tislelizumab followed by tislelizumab, one studied chemo-toripalimab followed by toripalimab, one focused on chemo-durvalumab followed by durvalumab, and one assessed chemo-pembrolizumab followed by pembrolizumab. In the two RCTs with neoadjuvant immunotherapy intervention, one investigated chemo-camrelizumab, and one examined chemo-nivolumab. The risk evaluated by the risk of bias assessment fell between allowable bounds (Supplementary Tables S2 and S3). Table 1 describes the main baseline features of the eight RCTs. Supplementary Table S4 shows the demographical dataset of the patients in the selected studies, while funnel plots and heterogeneity analyses are displayed in Supplementary Figures S1 and S2. After analysis, the I^2^ values for all studies were <50%, indicating no significant heterogeneity among the included studies.

### 3.2. Network Meta-Analysis

#### 3.2.1. Comparison of MPR and pCR

**Figure 2:**
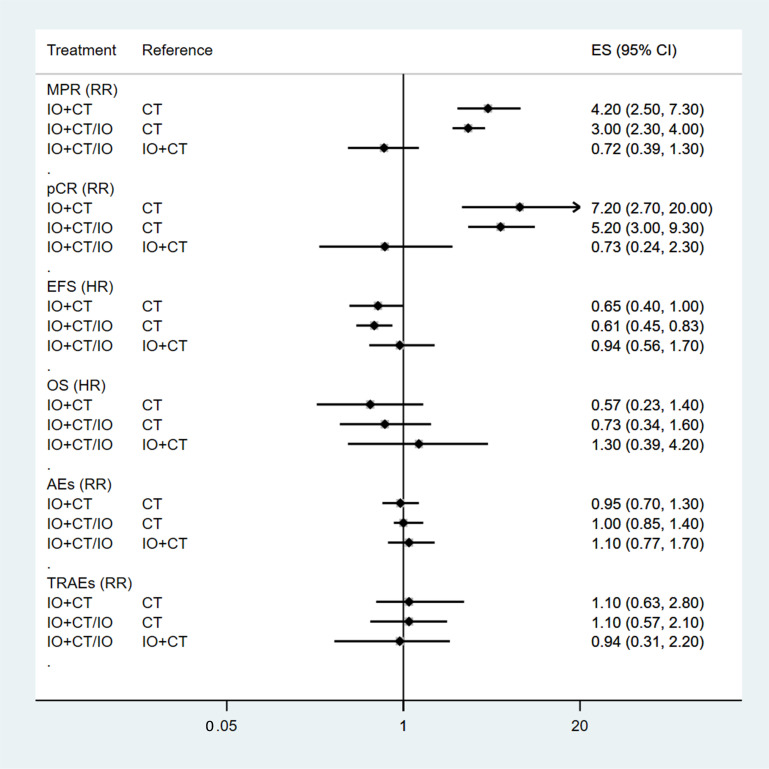
Forest plots for MPR, pCR, EFS, OS, AEs, and TRAEs. IO + CT: neoadjuvant chemoimmunotherapy; CT: neoadjuvant chemotherapy; IO + CT/IO: perioperative chemoimmunotherapy.

**Figure 3:**
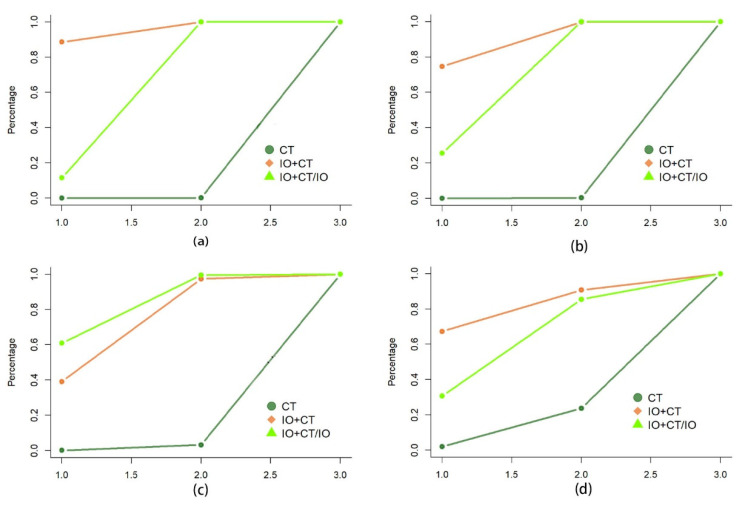
Treatment **rank probability curves** for MPR (**a**), pCR (**b**), EFS (**c**), and OS (**d**). IO + CT: neoadjuvant chemoimmunotherapy; CT: neoadjuvant chemotherapy; IO + CT/IO: perioperative chemoimmunotherapy.

The network plot illustrates the comparisons made between each therapy (Supplementary Figure S3). The relative risk (RR) of MPR was 4.2 (95% CI 2.5–7.3) for immunotherapy plus chemotherapy (IO + CT) versus chemotherapy (CT) and 3.0 (95% CI 2.3–4.0) for IO + CT/IO vs. CT, while for pCR, it was 7.2 (95% CI 2.7–20.0) for IO + CT versus CT and 5.2 (95% CI 3.0–9.3) for IO + CT/IO versus CT (Figure 2). The Bayesian ranking method used curves to show the likelihood of each therapy being ordered from first to last based on the RR and 95% CI. Neoadjuvant immunochemotherapy demonstrated the greatest potential to achieve the highest rankings in both MPR and pCR (Figure 3a,b). It is important to note that, while ranking provides an overview of relative efficacy, it does not necessarily indicate statistical significance between treatments.

#### 3.2.2. Comparison of EFS

The HRs of EFS among those with NSCLC who were treated with neoadjuvant immunochemotherapy, perioperative immunotherapy, or chemotherapy are displayed in Figure 2. Although no significant advantages were observed in EFS (HR 0.95, 95% CI 0.56–1.7) between perioperative immunochemotherapy and neoadjuvant immunochemotherapy, the Bayesian ranking results showed that there was a similar likelihood of neoadjuvant immunochemotherapy or perioperative immunochemotherapy being ranked first (Figure 3c). The findings indicate that patients with NSCLC could derive similar clinical benefit if they received either neoadjuvant immunochemotherapy or perioperative immunochemotherapy.

#### 3.2.3. Comparison of OS

The HR for perioperative immunochemotherapy compared to neoadjuvant immunochemotherapy in OS was 1.3 (95% CI 0.39–4.20) (Figure 2). A significant difference was not observed between these two interventions.

#### 3.2.4. Comparison of AEs and TRAEs

There was no notable distinction seen between perioperative immunochemotherapy and neoadjuvant immunochemotherapy in terms of AEs, TRAEs, and AEs and TRAEs of grade 3 or higher (Supplementary Figure S4).

### 3.3. Subgroup Analysis of EFS

#### 3.3.1. PD-L1 Expression (<1%, 1–49%, ≥50%)

**Figure 4:**
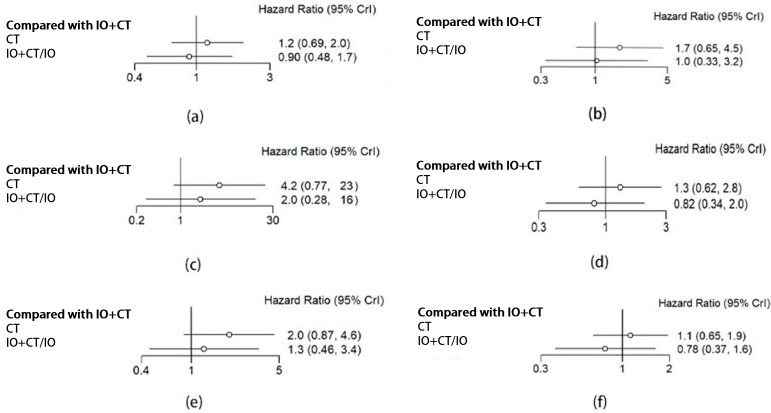
Forest plots for PD-L1 < 1 (**a**), PD-L1 1–49% (**b**), PD-L1 ≥ 50% (**c**), non-squamous NSCLC (**d**), squamous NSCLC (**e**), and pCR (**f**). IO + CT: neoadjuvant chemoimmunotherapy; CT: neoadjuvant chemotherapy; IO + CT/IO: perioperative chemoimmunotherapy.

**Figure 5:**
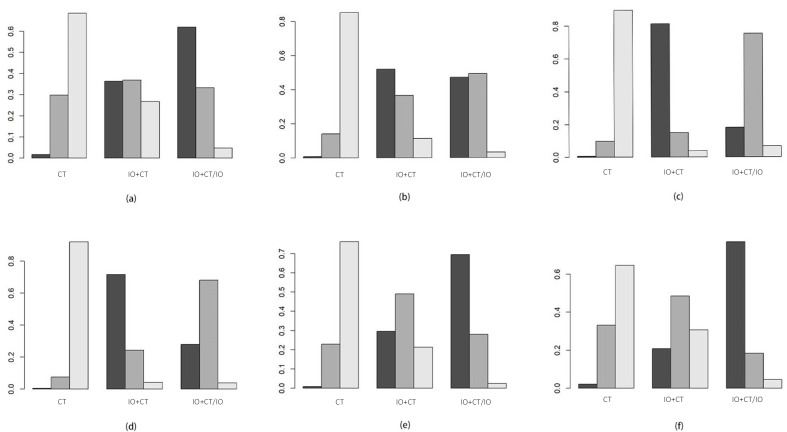
Bayesian ranking results for PD-L1 < 1 (**a**), PD-L1 1–49% (**b**), PD-L1 ≥ 50% (**c**), non-squamous NSCLC (**d**), squamous NSCLC (**e**), and pCR (**f**). IO + CT: neoadjuvant chemoimmunotherapy; CT: neoadjuvant chemotherapy; IO + CT/IO: perioperative chemoimmunotherapy.

Among those patients suffering from NSCLC who showed varying levels of PD-L1 expression, there was no significant difference in EFS. The HRs for EFS were as follows: PD-L1 < 1%: 0.90 (95% CI 0.48–1.7); PD-L1 1–49%: 1.0 (95% CI 0.33–3.2); PD-L1 ≥ 50%: 2.0 (95% CI 0.28–16.0) (Figure 4a–c). However, the Bayesian ranking results showed a difference in EFS survival. In the PD-L1 < 1% group, perioperative immunochemotherapy was the most promising therapy, being ranked first in EFS, while neoadjuvant immunochemotherapy showed an advantage in the PD-L1 ≥ 50% group. The efficacy of either neoadjuvant immunochemotherapy or perioperative immunochemotherapy produced similar benefits in the PD-L1 1–49% group (Figure 5a–c).

#### 3.3.2. Histological Type (Non-Squamous/Squamous)

The difference in EFS between neoadjuvant immunochemotherapy and perioperative immunochemotherapy was not significant in patients with non-squamous NSCLC and squamous NSCLC (HR: non-squamous 0.82, 95% CI 0.34–2.0; squamous 1.3, 95% CI 0.46–3.4) (Figure 4d,e). Nonetheless, the Bayesian ranking results showed that neoadjuvant immunochemotherapy was the most promising therapy for EFS in the non-squamous group, while perioperative immunochemotherapy was favored in the squamous group (Figure 5d,e).

#### 3.3.3. Reached pCR or Not (pCR/Non-pCR)

An analysis was performed to determine the impact of an additional adjuvant component in combination with neoadjuvant treatment on the survival rate, specifically looking at the EFS of two treatment regimens based on pCR status. This analysis found no significant difference in EFS between neoadjuvant immunochemotherapy and perioperative immunochemotherapy approaches for patients with NSCLC who did not achieve a pCR (HR 0.78, 95% CI 0.37–1.6) (Figure 4f). However, the Bayesian ranking results indicated that perioperative immunochemotherapy was the most promising therapy for EFS in the non-pCR group (Figure 5f). The pCR group could not be analyzed due to incomplete primary data.

#### 3.3.4. Others

In the subgroup analyses based on age, and ECOG performance-status score, no significant difference was observed in EFS (Supplementary Figure S5) (HR: age <65 1.1, 95% CI 0.39–2.9; age ≥ 65 0.95, 95% CI 0.45–2.0; ECOG = 0 1.1, 95% CI 0.4–2.9; ECOG = 1 1.1, 95% CI 0.44–2.7;). However, the Bayesian ranking results indicated that neoadjuvant immunochemotherapy was the most promising therapy for EFS in males, non-smokers, and the population who used carboplatin (Supplementary Figure S6).

## 4. Discussion

The network meta-analysis found that there were no statistically significant differences between perioperative immunotherapy and neoadjuvant immunotherapy in terms of MPR, pCR, EFS, and OS. Based on the subgroup analyses, neoadjuvant immunotherapy may have advantages over perioperative immunotherapy in PD-L1 high-expression patients, non-squamous cell carcinoma patients as well as non-smoking patients. However, in the PD-L1 low- or negative-expression cohort, squamous cell carcinoma cohort, and non-pCR patients, perioperative immunotherapy may offer better outcomes. These results could provide important evidence for personalized treatment of non-small cell lung cancer.

Upon comprehensive analysis, we observed some differences in clinical outcomes between perioperative immunotherapy and neoadjuvant immunotherapy in the Bayesian rank analysis; however, these did not reach statistical significance in the forest analysis. This may be due to the relatively small number of relevant studies reported so far as we were only able to include eight RCT studies for analysis, among which only two adopted neoadjuvant alone. The limited sample size of patients may contribute to the current conclusions reached. Furthermore, most perioperative studies are immature and longer follow-up times are still needed to be able to see the real benefit of adjuvant treatments.

Considering the EFS survival according to PD-L1 expression, PD-L1-negative patients did better with perioperative treatment than with the neoadjuvant regime alone. Previous studies showed that neoadjuvant chemoimmunotherapy increases tumor immune lymphocytes’ infiltration in the stroma area [23]. We formed the hypothesis that such effects might be insufficient for PD-L1-negative patients to induce immune memory and benefit from sustained checkpoint inhibitors after surgery. However, the current findings are just drawn from a meta-analysis, and only three of the eight studies included were RCTs reporting relevant outcomes, with 797 cases receiving perioperative immunotherapy, which was far more than the only 179 cases treated with neoadjuvant immunotherapy alone. Adopting uniform definitions (e.g., PD-L1 thresholds) across future trials would help further meta-analytic consistency. All these factors may lead to the EFS difference observed here. Future efforts should be made to analyze the tumor immune microenvironment of patients to better understand which individuals are likely to have an immune system that will respond to the additional checkpoint inhibitors after surgery.

Considering the subgroups of patients achieving pCR or not, Bayesian ranking results showed that the perioperative setting resulted in more impressive EFS survival than neoadjuvant immunotherapy alone. This suggests that patients who did not achieve pCR may experience survival advantages from the adjuvant component of treatment, particularly when considering the continuation of immunotherapy after surgery. However, only CM816, CM77T and KN671 were included in this analysis, and the limited sample size might be the reason why such differences were not observed in the forest plot. In conclusion, patients in the following three subgroups—the PD-L1 < 1% group, the non-squamous group, and the non-pCR group—may potentially derive greater benefit from subsequent adjuvant treatment. Additionally, patients with high TMB and those with lymph node involvement, particularly those with N2 stage metastasis, may also derive greater benefit from adjuvant therapy [24,25]. However, these findings are exploratory, and further prospective trials are required to determine whether adjuvant immunotherapy may provide further advantages for patients after receiving neoadjuvant chemoimmunotherapy. Aside from pCR or MPR, future work should focus on exploring the association between RVT and survival, and we have already seen the preliminary results from CM816. Regarding distinct levels of remaining illness, various results have been observed, with two-year event-free survival rates of 90%, 60%, 57%, and 39% for patients with residual disease volumes of 0–5%, >5–30%, >30–80%, and >80%, respectively. Each 1% RVT is associated with a 0.017 rise in the hazard ratio for EFS. Non-pCR is still a wide spectrum population, and not all these patients can benefit to the same extent from that additional 1 year of adjuvant treatment. Hence, more effective biomarkers are warranted to identify which patients could potentially benefit from adjuvant treatment in addition to neoadjuvant treatment.

A meta-analysis published in 2023 conducted an exploratory analysis of PFS/EFS and OS for perioperative immunotherapy versus neoadjuvant immunotherapy. This study revealed that perioperative immunotherapy did not significantly alter EFS/PFS and OS compared to neoadjuvant immunotherapy (EFS/PFS RR 0.83, 95% CI 0.57–1.21; OS RR 1.08, 95% CI 0.59–1.98) [26]. This finding was corroborated by the present study. In addition, we performed extra subgroup analyses to investigate the influence of PD-L1 expression profile, histologic type, and attainment of pCR. This meta-analysis included all eight relevant RCTs, rendering it the most comprehensive NMA undertaken to date.

It is important to recognize that this study has various limitations. Initially, our meta-analysis was based on published findings rather than individual patient data, which could have potentially introduced biases. Additionally, the limited number of eligible studies in the neoadjuvant chemoimmunotherapy group (only two studies with 406 patients) may have introduced potential biases, emphasizing the need for more studies to validate the accuracy of the results. Second, the immune checkpoint inhibitors used in different studies varied, with only one or two RCTs using each drug, and many studies are not yet fully mature (particularly for OS). As a result, the analysis of outcomes relied more heavily on MPR and pCR, which could have influenced the outcomes. Furthermore, during the analysis, we considered the placebo cohort as the control and the chemotherapy-only cohort as the chemotherapy treatment, which might have affected the comparability of the interventions. Another limitation is the variation in follow-up durations across studies, which could have influenced the observed outcomes. Finally, there was a lack of studies directly comparing neoadjuvant immunotherapy with perioperative immunotherapy, which limited the strength of evidence emerging from the meta-analysis. Future direct head-to-head RCTs or robust real-world evidence are recommended. Studies with standardized methodologies and direct comparisons are warranted to address these limitations and provide more robust evidence.

## 5. Conclusions

In conclusion, our NMA suggests that there is no substantial disparity in overall effectiveness between receiving neoadjuvant immunotherapy and receiving perioperative immunotherapy in patients with resectable NSCLC, yet this finding is also tempered by the small sample size of neoadjuvant-only RCTs. However, the findings on potential differences in PD-L1-negative patients (or non-pCR patients) are exploratory. Further investigation is needed to explore whether the neoadjuvant immunotherapy modality confers advantages in the PDL1 high-expressing cohort, squamous lung cancer cohort, and non-smoking cohort, or whether perioperative immunotherapy can provide additional benefits for the PD-L1 low-expressing cohort, squamous lung cancer cohort and those who have not reached pCR. The inclusion of additional RCTs in the future is warranted to address these questions comprehensively. Nevertheless, our findings provide crucial proof to guide the management of individuals with resectable NSCLC.

## Figures and Tables

**Table 1:** Characteristics of included studies.

Trial	Author, Year	Phase	NCT	Clinical Stage	No. of Patients	Neoadjuvant	No. of Cycles	Adjuvant	No. of Cycles	Endpoint(s)	Median Follow-Up Time (Months)	Immunotherapy Agent
NADIM II	Provencio, 2023; Provencio, 2022	II, 2:1; Open-label	NCT03838159	IIIA-IIIB **	57	CT + nivo	3	nivo	6	MPR, pCR, OS, AEs, TRAEs	26.1 (17.4–30.9)	IO + CT/IO vs. CT
					29	CT	3					
RATIONALE-315	Yue, 2023	III, 1:1	NCT04379635	II-IIIA **	226	CT + tis	3 or 4	tis	8	MPR, pCR	16.8	IO + CT/IO vs. CT
					227	CT + pbo	3 or 4	pbo	8			
NEOTORCH	Lu, 2023	III, 1:1	NCT04158440	II-IIA-IIIB **	202	CT + tori	3	tori	13	MPR, pCR, EFS, OS	18.3	IO + CT/IO vs. CT
					202	CT + pbo	3	pbo	13			
CheckMate 77T	Cascone, 2023	III, 1:1	NCT04025879	II-IIIB **	229	CT + nivo	4	nivo	1y	MPR, pCR, EFS, AEs	15.7	IO + CT/IO vs. CT
					232	CT + pbo	4	pbo	1y			
AEGEAN	Heymach, 2023	III, 1:1	NCT03800134	IIA-IIIB **	400	CT + durva	4	durva	12	MPR, pCR, EFS, AEs, TRAEs	11.7 (0.0–46.1)	IO + CT/IO vs. CT
					402	CT + pbo	4	pbo	12			
KEYNOTE-671	Wakelee, 2023	III, 1:1	NCT03425643	IIA-IIIB **	397	CT + pembro	4	pembro	13	MPR, pCR, EFS, OS, TRAEs	25.2 (7.5–50.6)	IO + CT/IO vs. CT
					400	CT + pbo	4	pbo	13			
TD-FOREKNOW	Lei, 2023	II, 1:1, Open-label	NCT04338620	IIIA-IIIB [T3N2M0] **	43	CT + cam	3			MPR, pCR, EFS, TRAEs	14.1 (9.2–20.9)	IO + CT vs. CT
					45	CT	3					
CheckMate 816	Forde, 2022; Forde, 2023	III, 1:1, Open-label	NCT02998528	IB-IIIA *	179	CT + nivo	3	CT/RT/CT + RT		MPR, pCR, EFS, OS, AEs, TRAEs	21.0	IO + CT vs. CT
					179	CT	3					

* AJCC 7th ed. ** AJCC 8th ed. AEs: adverse events; CT: chemotherapy; cam: camrelizumab; durva: durvalumab; EFS: event-free survival; IO + CT: neoadjuvant chemoimmunotherapy; IO + CT/IO: perioperative chemoimmunotherapy; MPR: major pathologic response; NCT: National Clinical Trials; nivo: nivolumab; OS: overall survival; pbo: placebo; pCR: pathologic complete response; pembro: pembrolizumab; RT: radio therapy; Tis: tislelizumab; tori: toripalimab; TRAEs: treatment-related adverse events; 1y: one year.

## Data Availability

The datasets generated during and/or analyzed during the current study are available from the corresponding author on reasonable request.

## Supplementary Materials

The following supporting information can be downloaded at: https://www.mdpi.com/article/10.3390/curroncol32030184/s1, Table S1. MEDLINE (PubMed) search terms (Search performed 27th November 2023); Table S2. EMBASE search terms; Table S3. CENTRAL (Cochrane) search terms; Table S4. Characteristics at Baseline; Figure S1. Funnel plots; Figure S2. Heterogeneity analyses; Figure S3. Network plot; Figure S4. Forest plots; Figure S5. Forest plots; Figure S6. Bayesian ranking results for EFS.
