# Supplementary material for: Efficacy and Safety of Perioperative Immunotherapy for Patients with Non-Small Cell Lung Cancer: A Systematic Review and Network Meta-Analysis
Source: Curr Oncol. 2025 Mar 20;32(3):184. doi: 10.3390/curroncol32030184 (PMC11940944; doi:10.3390/curroncol32030184)
Supplement: Supplementary file 1 [file curroncol-32-00184-s001.zip › curroncol-3392432-supplementary.pdf]

Supplementary Appendix

TABLE OF CONTENTS

SUPPLEMENTARY TABLES 2

SUPPLEMENTARY FIGURES 7

SUPPLEMENTARY TABLES

Supplements Table S1. MEDLINE (PubMed) search terms (Search performed 27th November 2023).

Search	Query	Items found
#4	#1 AND #2 AND #3	748
#3	"Neoadjuvant Therapy"[MeSH] OR neoadjuvant[tiab] OR "neo-adjuvant"[tiab] OR perioperative[tiab] OR preoperative[tiab] OR "peri-operative"[tiab] OR "pre-operative"[tiab]	497,443
#2	Immunotherapy[MeSH] OR "Antibodies, Monoclonal, Humanized"[MeSH] OR "Programmed Cell Death 1 Receptor"[MeSH] OR "CTLA-4 Antigen"[MeSH] OR "B7-H1 Antigen"[MeSH] OR "Immune Checkpoint Inhibitors"[MeSH] OR immunotherap*[tiab] OR immuno-therap*[tiab] OR immunetherap*[tiab] OR immune-therap*[tiab] OR immune checkpoint inhibit*[tiab] OR nivolumab[tiab] OR ipilimumab[tiab] OR sintilimab[tiab] OR durvalumab[tiab] OR atezolizumab[tiab] OR pembrolizumab[tiab] OR avelumab[tiab] OR tremelimumab[tiab] OR camrelizumab[tiab] OR tislelizumab[tiab] OR toripalimab[tiab]	531,722
#1	"Carcinoma, Non-Small-Cell Lung"[MeSH] OR non-small-cell lung carcinoma*[tiab] OR non-small-cell lung cancer*[tiab] OR non-small cell lung cancer*[tiab] OR non-small cell lung carcinoma*[tiab] OR nonsmall cell lung cancer*[tiab] OR nonsmall cell lung carcinoma*[tiab] OR NSCLC[tiab]	104,074

Abbreviations: [MeSH] = Medical Subject Headings; [tiab] = Title/Abstract

Supplements Table S2. EMBASE search terms.

Search	Query	Items found
#4	#1 AND #2 AND #3	2,108
#3	'neoadjuvant therapy'/exp OR ‘neoadjuvant’:ab,ti,kw OR 'neo-adjuvant':ab,ti,kw OR ‘perioperative’:ab,ti,kw OR ‘preoperative’:ab,ti,kw OR 'peri-operative':ab,ti,kw OR 'pre-operative':ab,ti,kw	733,045
#2	'immunotherapy'/exp OR 'monoclonal antibody'/exp OR 'immune checkpoint inhibitor'/exp OR 'programmed death 1 receptor'/exp OR 'programmed death 1 ligand 1'/exp OR 'cytotoxic t lymphocyte antigen 4'/exp OR ‘immunotherap*’:ab,ti,kw OR 'immuno-therap*':ab,ti,kw OR ‘immunetherap*’:ab,ti,kw OR 'immune-therap*':ab,ti,kw OR 'immune checkpoint inhibit*':ab,ti,kw OR ‘nivolumab’:ab,ti,kw OR ‘ipilimumab’:ab,ti,kw OR ‘sintilimab’:ab,ti,kw OR ‘durvalumab’:ab,ti,kw OR ‘atezolizumab’:ab,ti,kw OR ‘pembrolizumab’:ab,ti,kw OR ‘avelumab’:ab,ti,kw OR ‘tremelimumab’:ab,ti,kw OR ‘camrelizumab’:ab,ti,kw OR ‘tislelizumab’:ab,ti,kw OR ‘toripalimab’:ab,ti,kw	1,122,952
#1	'non small cell lung cancer'/exp OR 'non-small-cell lung cancer*':ab,ti,kw OR 'non-small-cell lung carcinoma*':ab,ti,kw OR 'non-small cell lung cancer*':ab,ti,kw OR 'non-small cell lung carcinoma*':ab,ti,kw OR 'nonsmall cell lung cancer*':ab,ti,kw OR 'nonsmall cell lung carcinoma*':ab,ti,kw OR ‘NSCLC’:ab,ti,kw	236,425

Abbreviations: /exp = explosion in Emtree terms; :ti,ab,kw = title, abstract, keywords

Supplements Table S3. CENTRAL (Cochrane) search terms.

Search	Query	Items found
#4	#1 AND #2 AND #3	257
#3	"Neoadjuvant Therapy"[MeSH] OR neoadjuvant[tiab] OR "neo-adjuvant"[tiab] OR perioperative[tiab] OR preoperative[tiab] OR "peri-operative"[tiab] OR "pre-operative"[tiab]	78,281
#2	Immunotherapy[MeSH] OR "Antibodies, Monoclonal, Humanized"[MeSH] OR "Programmed Cell Death 1 Receptor"[MeSH] OR "CTLA-4 Antigen"[MeSH] OR "B7-H1 Antigen"[MeSH] OR "Immune Checkpoint Inhibitors"[MeSH] OR immunotherap*[tiab] OR immuno-therap*[tiab] OR immunetherap*[tiab] OR immune-therap*[tiab] OR immune checkpoint inhibit*[tiab] OR nivolumab[tiab] OR ipilimumab[tiab] OR sintilimab[tiab] OR durvalumab[tiab] OR atezolizumab[tiab] OR pembrolizumab[tiab] OR avelumab[tiab] OR tremelimumab[tiab] OR camrelizumab[tiab] OR tislelizumab[tiab] OR toripalimab[tiab]	27,773
#1	"Carcinoma, Non-Small-Cell Lung"[MeSH] OR non-small-cell lung carcinoma*[tiab] OR non-small-cell lung cancer*[tiab] OR non-small cell lung cancer*[tiab] OR non-small cell lung carcinoma*[tiab] OR nonsmall cell lung cancer*[tiab] OR nonsmall cell lung carcinoma*[tiab] OR NSCLC[tiab]	18,025

Abbreviations: [MeSH] = Medical Subject Headings; [tiab] = Title/Abstract

Supplements Table S4. Characteristics at Baseline.

Characteristic	NADIM II		AEGEAN		KEYNOTE 671		TD-FOREKNOW		CheckMate 816	
	IO+CT (N=57)	CT (N=29)	IO+CT (N=336)	CT (N=374)	IO+CT (N=397)	CT (N=400)	IO+CT (N=43)	CT (N=45)	IO+CT (N=179)	CT (N=179)
Age										
Median (range) — yr	65 (58–70)	63 (57–66)	65 (30–88)	65 (39–85)	63 (26–83)	64 (35–81)	61 (54-65)	61 (54-65)	64 (41–82)	65 (34–84)
Sex — no. (%)										
Male	36 (67)	16 (55)	252(68.9)	278(74.3)	279(70.3)	284(71.0)	34(79.1)	40(88.9)	128(71.5)	127(70.9)
Female	21 (37)	13 (45)	114(31.1)	96 (25.7)	118(29.3)	116 (29)	9 (20.9)	5 (11.1)	51 (28.5)	52 (29.1
ECOG performance-status score — no. (%)										
0	31 (54)	16 (55)	251(68.6)	255(68.2)	253(63.7)	246(61.5)	41(95.3)	43(95.6)	124(69.3)	117(65.4)
1	26 (46)	13 (45)	115(31.4)	119(31.8)	144(36.3)	154(38.5)	2 (4.7)	2 (4.4)	55 (30.7)	62 (34.6)
Geographic region — no. (%)										
Asia	—	—	142(38.8)	163(43.6)	123(31.0)	121(30.2)	—	—	85 (47.5)	92 (51.4)
Europe	—	—	141(38.5)	140(37.4)	—	—	—	—	41 (22.9)	25 (14.0)
North America	—	—	43 (11.7)	43 (11.5)	—	—	—	—	41 (22.9)	50 (27.9)
South America	—	—	40 (10.9)	28 (7.5)	—	—	—	—	12 (6.7)	12 (6.7)
Other	—	—	—	—	274(69.0)	279(69.8)	—	—	—	—
Smoking status — no. (%)										
Never	5 (9)	0	51 (13.9)	56 (15.0)	54 (13.6)	47 (11.8)	12 (27.9)	8 (17.8)	19 (10.6)	20 (11.2)
Former or current smoker	52 (91)	29(100)	315(86.1)	318(85.0)	343(86.4)	353(88.3)	31 (72.1)	37 (82.2)	160(89.4)	158(88.3)
Disease stage — no. (%)										
IB or II	—	—	—	—	—	—	—	—	65 (36.3)	62 (34.6)
II	—	—	104(28.4)	110(29.4)	118(29.7)	121(30.2)	—	—	—	—
IIIA	44 (77)	24 (83)	173(47.3)	165(44.1)	217(54.7)	225(56.2)	30 (69.8)	36 (80.0)	113(63.1)	115(64.2)
IIIB	13 (23)	5 (17)	88 (24.0)	98 (26.2)	62 (15.6)	54 (13.5)	13 (30.2)	9 (20.0)	—	—

TNM classification, primary tumor—no. (%)										
T1	12 (21)	4 (14)	44 (12.0)	43 (11.5)	55 (13.9)	61 (15.2)	2 (4.7)	4 (8.9)	—	—
T2	16 (28)	7 (24)	97 (26.5)	108(28.9)	106(26.7)	126(31.5)	19 (44.2)	18 (40.0)	—	—
T3	15 (26)	6 (21)	128(35.0)	129(34.5)	121(30.5)	109(27.2)	16 (37.2)	13 (28.9)	—	—
T4	14 (25)	12 (41)	97 (26.5)	94 (25.1)	115(29.0)	104(26.0)	6 (13.9)	10 (22.2)	—	—
TNM stage, regional lymph nodes — no. (%)										
N0	6 (11)	9 (31)	110(30.1)	102(27.3)	148(37.3)	142(35.5)	5 (11.6)	5 (11.1)	—	—
N1	10 (18)	4 (14)	75 (20.5)	87 (23.3)	81 (20.4)	71 (17.8)	4 (9.3)	9 (20.0)	—	—
N2	41 (72)	16 (55)	181(49.5)	185(49.5)	168(42.3)	187(46.8)	34 (70.1)	31 (68.9)	—	—
TNM stage, regional lymph nodes — no. (%)										
Squamous	21 (37)	14 (48)	169(46.2)	191(51.1)	226(56.9)	227(56.8)	27 (62.8)	32 (71.1)	87 (48.6)	95 (53.1)
Nonsquamous	36 (63)	15 (52)	196(53.6)	179(47.9)	171(43.1)	173(43.2)	16 (37.2)	13 (28.8)	92 (51.4)	84 (46.9)
PD-L1 expression — no. (%)										
Tumor cell <1%	—	—	122(33.3)	125(33.4)	138(34.8)	151(37.8)	7 (16.3)	8 (17.8)	78 (43.6)	77 (43.0)
Tumor cell 1 to 49%	—	—	135(36.9)	142(38.0)	127(32.0)	115(28.8)	—	—	51 (28.5)	47 (26.3)
Tumor cell ≥50%	—	—	109(29.8)	107(28.6)	132(33.2)	134(33.5)	—	—	47 (26.3)	42 (23.5)
Tumor cell ≥1	—	—	244(66.7)	249(66.6)	259(65.2)	249(62.3)	16 (37.2)	11 (24.4)	89 (49.7)	89 (49.7)
Unknown	—	—	—	—	—	—	20 (46.5)	26 (57.8)	12 (6.7)	13 (7.3)

Eastern Cooperative Oncology Group (ECOG) performance-status scores range from 0 to 5, with higher scores indicating greater disability. § Race was reported by the patients. **Patients' disease stage** according to the eighth edition of the AJCC Cancer Staging Manual were enrolled. CT: chemotherapy; IO+CT: neoadjuvant chemoimmunotherapy; IO+CT/IO: perioperative chemoimmunotherapy.

SUPPLEMENTARY FIGURES

Supplements Figure S1. Funnel plots.

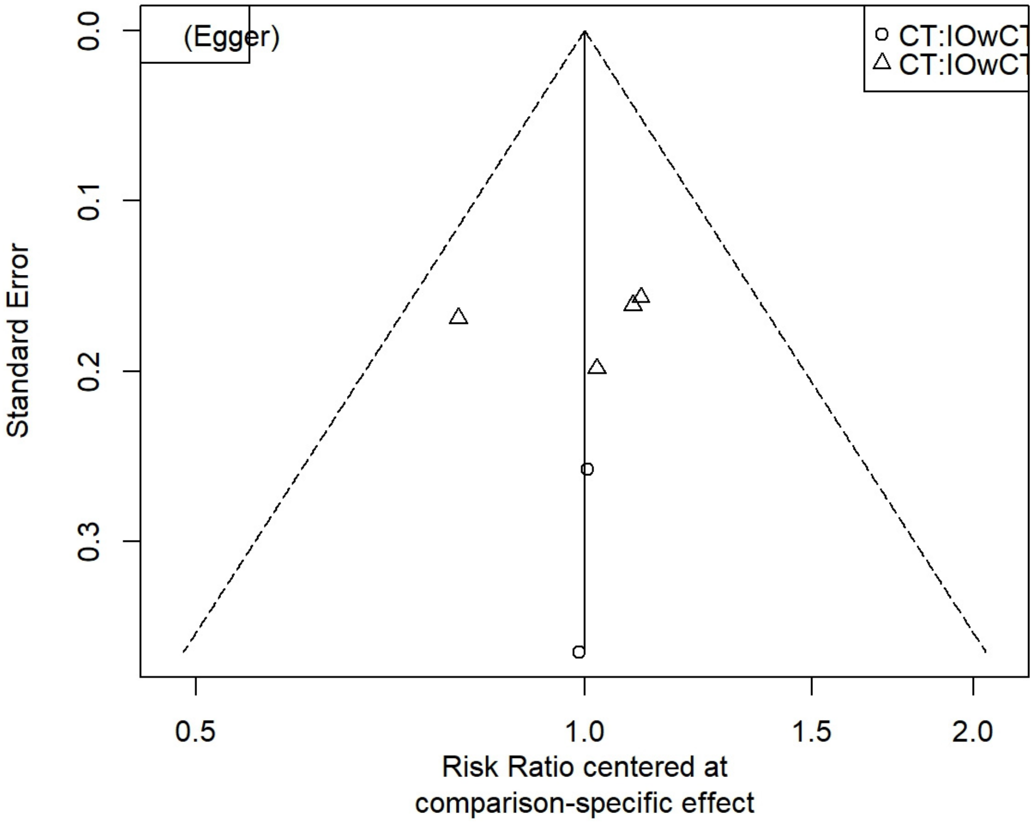

Supplements Figure S2. Heterogeneity analyses.

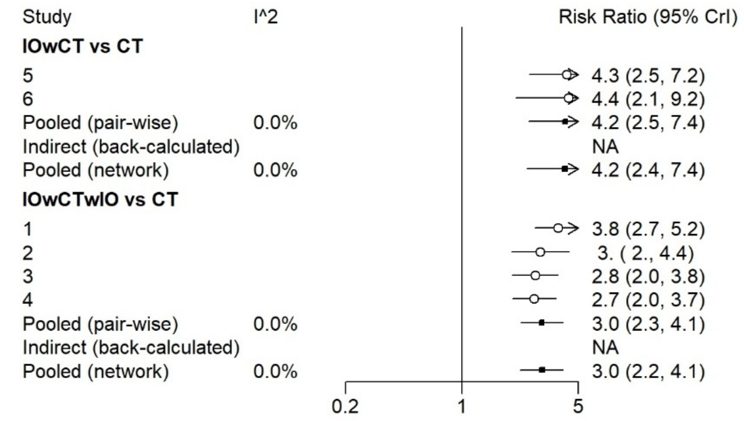

(a) MPR

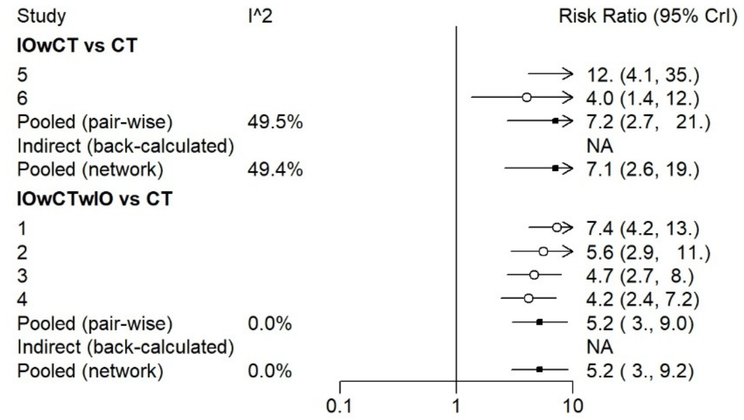

(b) pCR

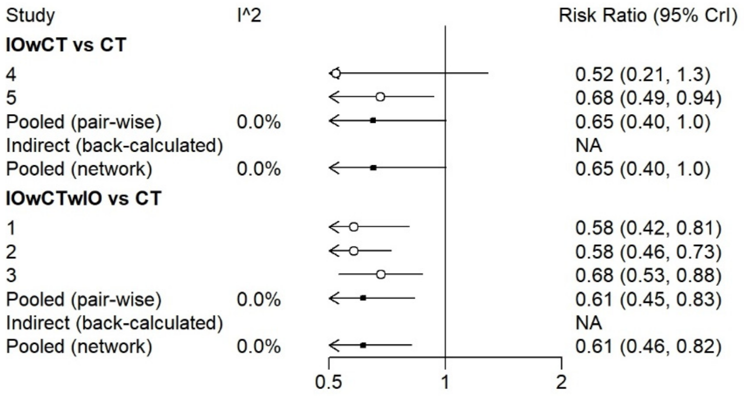

(c)EFS

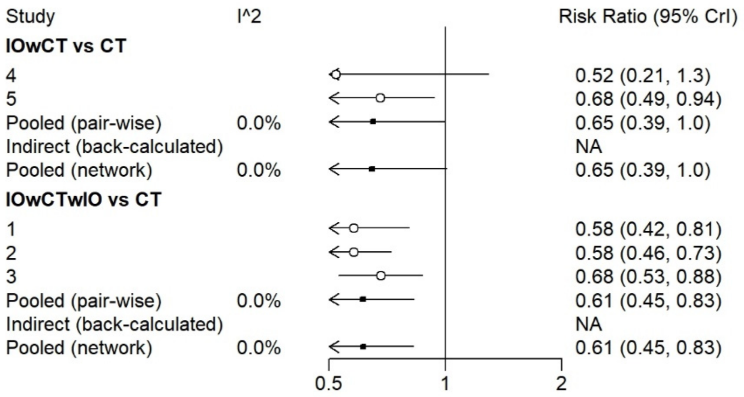
Study
I^2
Risk Ratio (95% CrI)
IOwCT vs CT
4
0.52 (0.21, 1.3)
5
0.68 (0.49, 0.94)
Pooled (pair-wise)
0.0%
0.65 (0.39, 1.0)
Indirect (back-calculated)
NA
Pooled (network)
0.0%
0.65 (0.39, 1.0)
IOwCTwIO vs CT
1
0.58 (0.42, 0.81)
2
0.58 (0.46, 0.73)
3
0.68 (0.53, 0.88)
Pooled (pair-wise)
0.0%
0.61 (0.45, 0.83)
Indirect (back-calculated)
NA
Pooled (network)
0.0%
0.61 (0.45, 0.83)
0.5
1
2

(d) OS

Supplements Figure S3. Network plot.

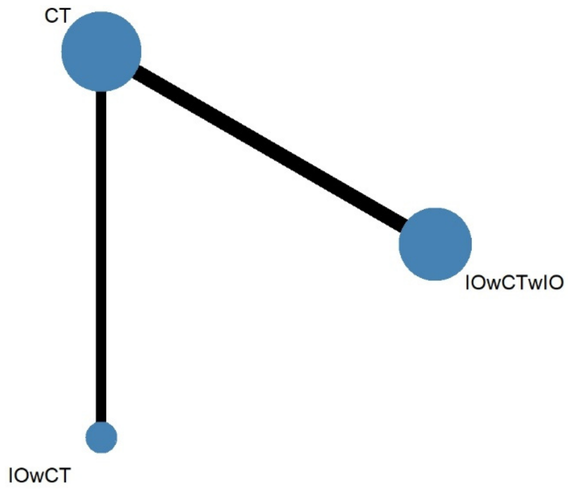

Supplements Figure S4. Forest plots.

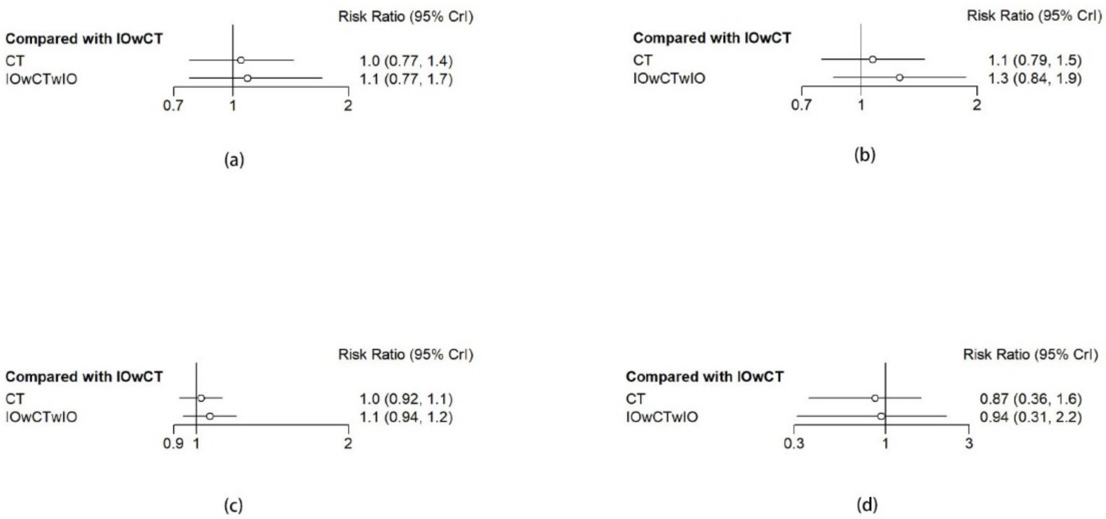

AEs (a), ≥3 AEs(b), TRAEs(c), and ≥3 TRAEs(d).

Supplements Figure S5. Forest plots.

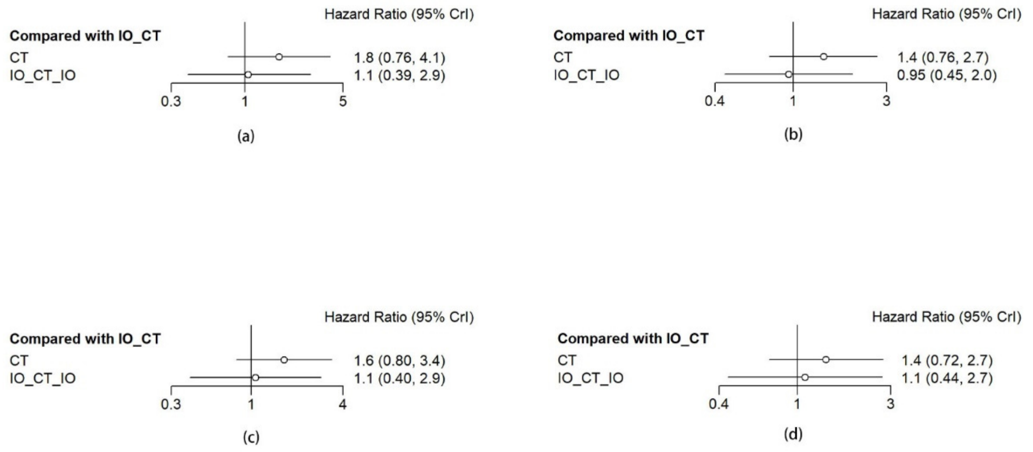

Age: ＜65 (a), ≥65 (b); ECOG: =0 (c), =1 (d).

Supplements Figure S6. Bayesian ranking results for EFS.

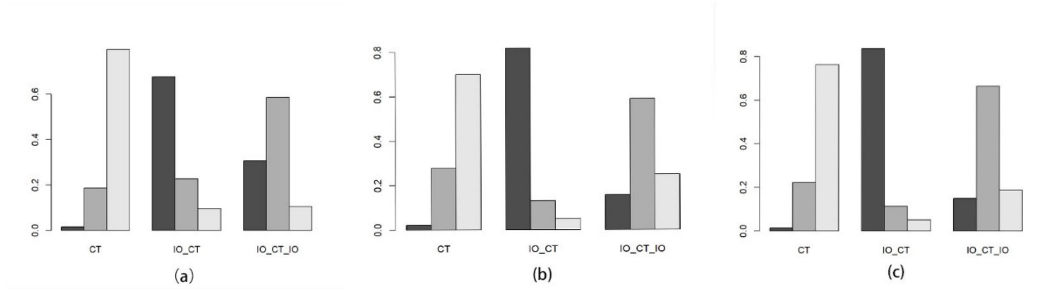

Males (a), non-smokers (b), the population who used carboplatin (c).
